# PLA1A expression as a diagnostic marker of BRAF-mutant metastasis in melanoma cancer

**DOI:** 10.1038/s41598-021-85595-7

**Published:** 2021-03-15

**Authors:** Gang Yang, Shuya Liu, Mazaher Maghsoudloo, Marzieh Dehghan Shasaltaneh, Parham Jabbarzadeh Kaboli, Cuiwei Zhang, Youcai Deng, Hajar Heidari, Maliheh Entezari, ShaoZhi Fu, QingLian Wen, Saber Imani

**Affiliations:** 1 Department of Oncology, The Affiliated Hospital of Southwest Medical University, Luzhou, Sichuan China; 2 Department of Oncology, Anyue Hospital of Traditional Chinese Medicine, Second Ziyang Hospital of Traditional Chinese Medicine, Ziyang, Sichuan China; 3 Laboratory of Systems Biology and Bioinformatics, Institute of Biochemistry and Biophysics, University of Tehran, Tehran, Iran; 4 Department of Genetics, Faculty of Advanced Science and Technology, Tehran Medical Sciences, Islamic Azad University, Tehran, Iran; 5 Department of Biology, Faculty of Science, University of Zanjan, Zanjan, Iran; 6 Department of Pharmacology, School of Pharmacy, Southwest Medical University, Luzhou, Sichuan China; 7 Graduate Institute of Biomedical Sciences, Research Center for Cancer Biology, and Center for Molecular Medicine, China Medical University, Taichung, Taiwan; 8 Department of Pathology, The Affiliated Hospital of Southwest Medical University, Luzhou, Sichuan China; 9 Institute of Materia Medical, College of Pharmacy, Army Medical University (Third Military Medical University), Chongqing, China

**Keywords:** Diagnostic markers, Skin cancer, Tumour biomarkers, Genetic markers

## Abstract

BRAF and NRAS are the most reported mutations associated to melanomagenesis. The lack of accurate diagnostic markers in response to therapeutic treatment in BRAF/NRAS-driven melanomagenesis is one of the main challenges in melanoma personalized therapy. In order to assess the diagnostic value of phosphatidylserine-specific phospholipase A1-alpha (PLA1A), a potent lysophospholipid mediating the production of lysophosphatidylserine, PLA1A mRNA and serum levels were compared in subjects with malignant melanoma (n = 18), primary melanoma (n = 13), and healthy subjects (n = 10). Additionally, the correlation between histopathological subtypes of BRAF/NRAS-mutated melanoma and PLA1A was analyzed. PLA1A expression was significantly increased during melanogenesis and positively correlated to disease severity and histopathological markers of metastatic melanoma. PLA1A mRNA and serum levels were significantly higher in patients with BRAF-mutated melanoma compared to the patients with NRAS-mutated melanoma. Notably, PLA1A can be used as a diagnostic marker for an efficient discrimination between naïve melanoma samples and advanced melanoma samples (sensitivity 91%, specificity 57%, and AUC 0.99), as well as BRAF-mutated melanoma samples (sensitivity 62%, specificity 61%, and AUC 0.75). Our findings suggest that PLA1A can be considered as a potential diagnostic marker for advanced and BRAF-mutated melanoma.

## Introduction

Although the incidence of malignant melanoma (MM) is less than 0.8% per 100,000 people, the mortality rate has significantly increased^1–3^. Indeed, over the past six decades, the overall mortality rate of MM has continuously increased by 6.5% per year in China^4,5^. Genetically, MM has one of the highest tumor mutation burden (TMB) across all solid tumors, with a mean mutation burden of over 20 mutations per megabase^6^. The mutations of the *B-Raf* proto-oncogene serine/threonine-kinase (*BRAF*), *NRAS*, and tumor protein p53 (*TP53*) are the main mutations that account for approximately 75% of all melanomas used for MM classification^7–9^. These mutations are associated with specific histopathological characteristics and melanomagenesis^10–12^. Melanoma patients with a defined TMB and carrying mutations in the cancer genome can benefit from early diagnosis^13–15^. An effective diagnostic strategy to identify BRAF/NRAS MM can allow the development of a subsequent targeted therapy that can improve the prognostic outcomes and therapeutic approaches among advanced MM patients^11,16–19^. The intra-tumoral heterogeneity of these genes leads to changes in the diagnostic and therapeutic strategies of melanoma, and personalized treatments and targeted therapies against melanoma have emerged^15,20^.

Our recent studies on multiple integrative and large-scale weighted gene co-expression network analysis identified phosphatidylserine-specific phospholipase A1-alpha (PS-PLA1A) as a novel network-based candidate marker in the diagnosis and prognosis of MM^21^. PS-PLA1 is a secreted cell membrane enzyme that is involved in catalyzing phosphatidylserine (PS) and 1-acyl-2-lysophosphatidylserine (lyso-PS) to hydrolyze fatty acids at the sn-1 position of these phospholipids. Recent studies showed that serum PLA1A levels are associated with tumor pathogenesis^22–24^, indicating its potential use as a diagnostic marker for monitoring several cancers including hepatocellular carcinoma^25^, gastric cancer^26^, colorectal cancer^27^, and melanoma^28^. PLA1A was identified as an individual marker in different genetically modified animals. PLA1A has been introduced PLA1A in laboratory medicine and in current clinical settings as an early-diagnostic metastatic and/or therapeutic marker^27^. mRNA and serum PLA1A levels in liquid biopsy diagnostics resulted promising tools for early diagnosis screening, tumor heterogeneity, and drug resistance of tumors with genomic mutations^15,29^.

Increasing mRNA and serum PLA1A levels are significantly associated with different clinical stages of melanoma, which suggests the potential involvement of the PS PLA1/lysophosphatidylserine axis in melanoma pathogenesis^22,28^. Despite numerous experimental studies, the diagnostic and prognostic values of PLA1A among different mutant melanoma patients and PLA1A role in melanomagenesis remain unknown. Certainly, effective diagnostic markers in the treatment of BRAF/NRAS-mutant melanoma are important in the development of a targeted therapy against advanced metastatic melanoma. Therefore, the aim of this study was at first to investigate the diagnostic value of PLA1A in melanoma patients at different stages and with different mutations to confirm the diagnostic value of PLA1A during melanomagenesis. Secondly, evidence in the use of PLA1A as a diagnostic and non-invasive therapeutic marker was documented to predict the clinical outcomes for advanced BRAF/NRAS-mutant MM.

## Results

### Clinicopathological findings

The demographic and clinicopathological features of the patients are summarized in Table 1. No differences between groups with regards to age and gender were found. A total of 41 patients were selected for this study according to the inclusion/exclusion criteria, who were then subdivided into three groups: 10 patients with naïve/normal melanoma (stage 0–I melanoma) diagnosed more than 2 years ago, 13 patients with primary melanoma (stage II–III melanoma) and 18 MM (stage III–IV melanoma). Among the 41 total patients, the genome of 22 (53%) females with a median age at diagnosis of 55 years was sequenced. Moreover, the melanomas were mostly isolated from the lower limb and hip (52%), as well as the trunk and neck (22%). The median thickness of melanoma was 2.2 mm in the primary and 5.4 mm in the metastatic group. Histologically, more than 54.8% (17/31) of melanoma patients were diagnosed through the pathological analysis of metastatic lymph nodes (67.8%) and metastasis in the lungs (19.4%). Furthermore, no patient was subjected to neoadjuvant therapy, and the 24% of melanomas patients underwent routine chemotherapy (Table 1).

**Table 1:** Demographic and baseline clinic-philological characteristics.

Variable	Control group	Melanoma patients	
	Naïve	Primary	Metastasis
**Demographic variables**			
Subject's n (%)	10 (24.4)	13 (31.7)	18 (43.9)**^,#^
Gender (F/M)	7/3	4/9	11/7
Age (years)	49.8 ± 8.2	52.5 ± 9.2	59.3 ± 10.5
**Histopathological variables**			
**Anatomic location n (%)**			
Face	0	0	2 (11.1)
Trunk and neck	5 (50)	3 (23.1)	1 (5.6)
Upper limb and shoulder	2 (20)	0	2 (11.1)
Lower limb and hip	3 (30)	9 (69.2)**	9 (50.0)**
NA	0	1 (7.7)	4 (22.2)
**T stage (thickness) n (%)**			
T2	–	2 (15.4)	9 (50.0)^##^
T3	–	2 (15.4)	3 (16.7)
T4	–	6 (46.1)	2 (11.1)^##^
TX	–	3 (23.1)	4 (22.2)
**N stage n (%)**			
N0	–	4 (30.8)	5 (27.8)
N1	–	0	7 (38.8)
N2	–	6 (46.1)	4 (22.2)
N3	–	1 (7.7)	1 (5.6)
NX	–	2 (15.4)	1 (5.6)
**M Stage n (%)**			
M0	–	11 (84.6)	3 (16.7)^##^
M1	–	2 (15.4)	15 (83.3)^##^
**Breslow n (%)**			
< 1 mm	–	2 (15.3)	0
1–2 mm	–	0	2 (11.1)
2.1–4.0 mm	–	7 (53.98)	2 (11.1)^#^
> 4 mm	–	3 (23.1)	10 (55.6)^##^
NXA	–	3 1 (723.71)	4 (22.2)
**Metastatic organ n (%)**			
Lymph nodes	–	8 (61.5)	10 (55.5)^#^
Fat and muscle	–	0	1 (5.6)
Lungs	–	4 (30.8)	4 (22.2)
Liver and gallbladder	–	0	2 (11.1)
NA	–	1 (7.7)	1 (5.6)
**Therapy variables**			
**Neoadjuvant therapy n (%)**			
No	10 (100)	13 (100)	18 (100)**^,#^
**Chemotherapy n (%)**			
Yes	0	2 (15.4)	8 (44.5)^##^
No	0	11 (84.6)	10 (55.5)

Data are presented as mean ± SD for all others. Naïve, healthy subjects with normal skin.

Patients were categorized according to the World Health Organization (WHO) guidelines and the pTNM Union for International Cancer Control (UICC) pathological staging criteria.

*NA* not available, *NX* not determined.

*p < 0.05 and **p < 0.001 vs the naïve group; ^#^*p* < 0.05 and ^##^*p* < 0.001 vs the primary group.

### PLA1A expression during melanogenesis

**Figure 1:**
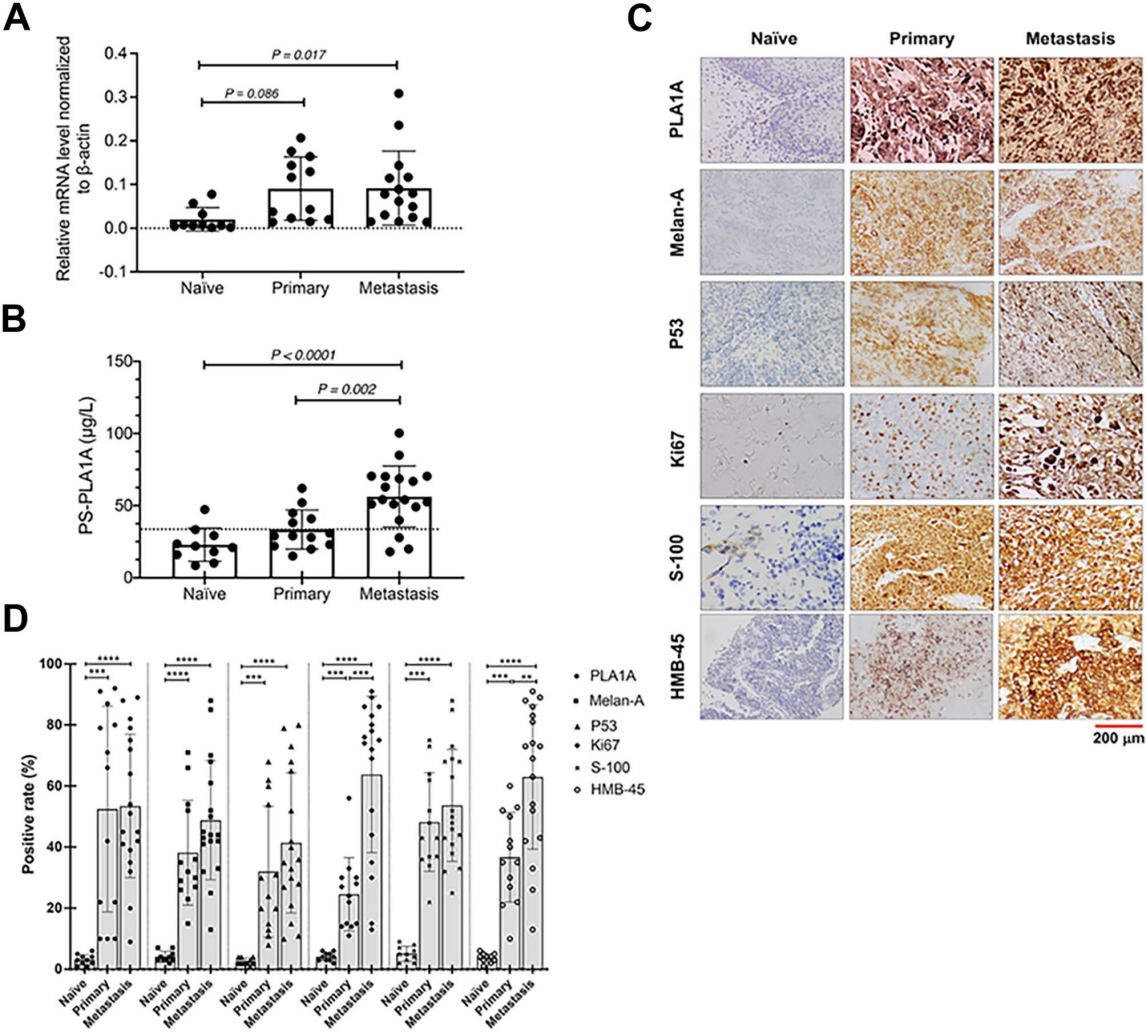
Analysis of PLA1A expression among different patient samples. (**A**) The expression of PLA1A in normal naïve/normal melanoma (n = 10), primary melanoma (n = 13) and metastatic melanoma (n = 18) tissues measured using TaqMan MicroRNA assays kit. (**B**) Comparison of serum PLA1A levels (PS-PLA1A) between naïve/normal melanoma (n = 10), primary melanoma (n = 13) and metastasis melanoma (n = 18). Representative histological staining imaging (**C**) and quantification of the relative expression (**D**) of an invasive and aggressive index of PLA1A, in comparison to the invasive panel, Ki-67+, S-100+, P53+, HMB-45+, and Melena + index of tumor sections in various groups. *p < 0.05 and **p < 0.001 vs the naïve group; ^#^*p* < 0.05 and ^##^*p* < 0.001 vs the primary group.

Previous screenings for PLA1 expression among different genders and stages of melanoma revealed that high serum PLA1A levels were associated with different clinical stages of melanoma in females^28^. Therefore, as a main member of PLA1 during melanogenesis, both PLA1A mRNA expression and serum levels were measured in our collected melanoma tissues and serum using quantitative Real-time PCR analysis (qRT-PCR) and enzyme-linked immunosorbent assay (ELISA), respectively. PLA1A mRNA expression and serum levels significantly increased during melanogenesis (Fig. 1). Interestingly, the average PLA1A expression was consistently and significantly higher in tissues of metastatic melanoma (n = 15) compared to naïve/control melanoma samples (n = 10). In addition, PLA1A levels were significantly higher in primary melanoma tissues (n = 12) compared to naïve/control melanoma samples (Fig. 1A). Furthermore, serum PLA1A levels were significantly increased in metastatic (56.27 ± 21.23 µg/L) and primary (33.47 ± 13.59 µg/L) group compared to the naïve group (22.93 ± 11.53 µg/L) (*p* ≤ 0.001) (Fig. 1B). The invasive and aggressive indexes of PLA1A were compared by immunohistochemistry, with the invasive panel including Ki-67^+^, S-100^+^, P53^+^, HMB-45^+^, and Melena^+^ index evaluated in tumor sections (Fig. 1C). As expected, the expression of all the invasive indexes was significantly higher in patients with MM compared to that in the naïve/control melanoma patients (Fig. 1D). As illustrated in Fig. 1C, the percentage of PLA1A positive cells was 53.44 ± 23.34% in the metastatic group, 52.46 ± 32.67% in the primary group and 3.21 ± 1.67% in the naïve group. Similarly, HMB-45, S-100 and Ki-67 invasive indexes were highly expressed in more than 50% of the metastatic patients (*p* < 0.001). However, no significant differences were observed in PLA1A expression between primary melanoma tissue and metastatic tissue. Notably, PLA1A was highly expressed in advanced metastatic melanoma and invasive melanoma samples, as well as Ki-67, and they had the same HMB-45, S-100, and Ki-67 invasive index. Since PLA1A can be positive in more than 50 cases that are HMB-45, S-100 and Ki-67 positive, it can be a useful component in the diagnosis of metastasis melanomas by immunohistochemistry. Thus, these findings revealed that PLA1A suppressed melanoma tumor proliferation and angiogenesis in the metastasis of human melanoma cancer. Regarding the ratio of mRNA to serum PLA1A, the expression of PLA1A significantly increased during melanogenesis. Therefore, a significant relationship exists between PLA1A levels and melanoma stage.

### Correlation of PLA1A expression with melanogenesis

**Figure 2:**
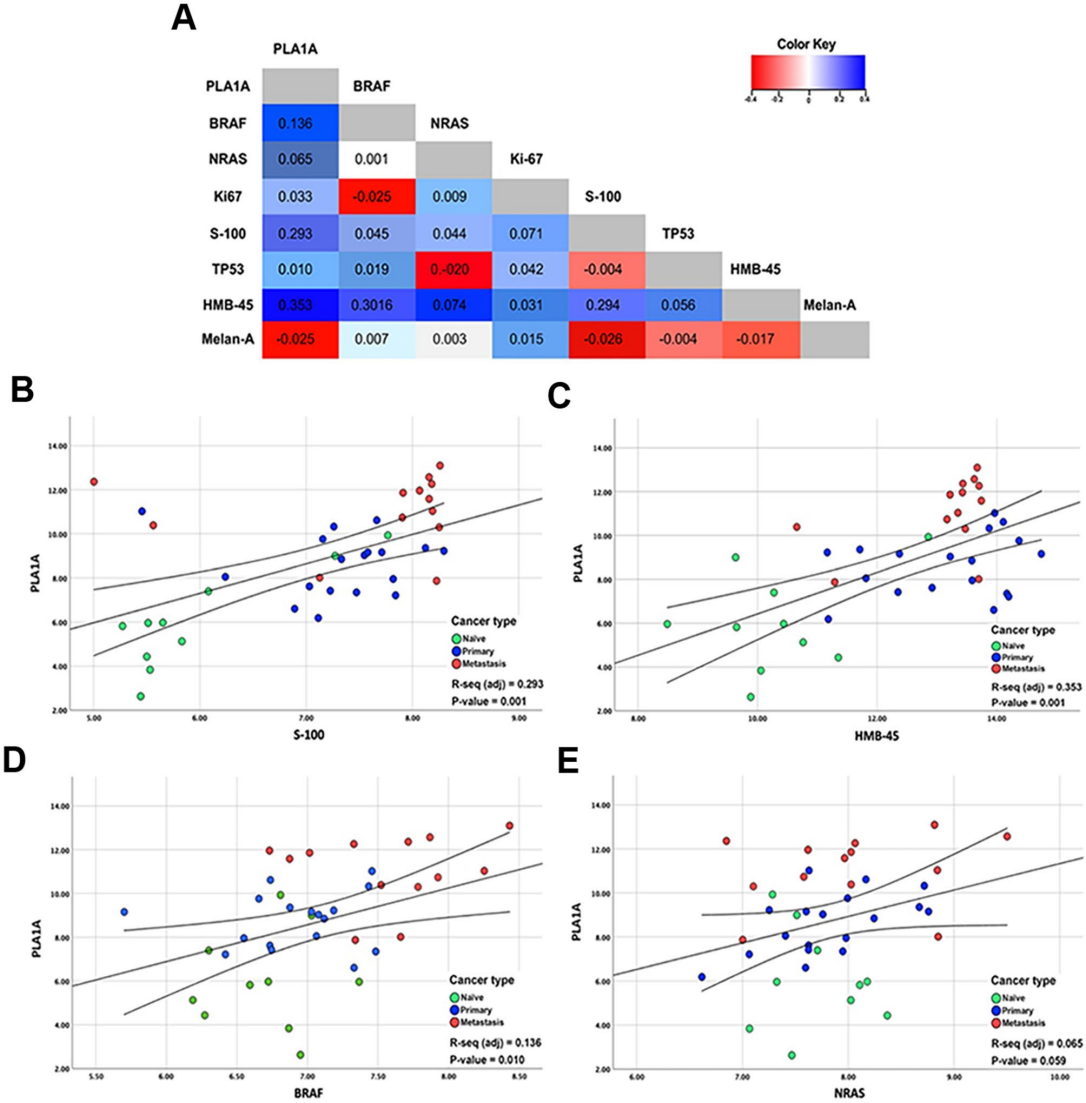
Correlation coefficient of PLA1A with diagnostic markers across metastatic melanoma. (**A**) Linear logistic regression analysis to predict the accuracy of PLA1A expression in comparison to the routine diagnostic markers of malignant melanoma. By considering both mRNA and serum levels, PLA1A was found as correlated to the expression of S-100 (**B**), HMB-45 (**B**), BRAF (**C**), and NRAS (**E**) in naïve/normal melanoma (green cycle), primary melanoma (blue cycle) and metastasis melanoma (red cycle).

The diagnosis of MM represents a challenge among a wide range of melanoma samples with different morphologies and immunohistochemical features. In order to verify whether PLA1A is associated with a type of diagnostic marker of MM, the linear logistic regression analysis was performed to predict the accuracy of the detection of PLA1A expression and characteristics of MM (Fig. 2). Figure 2A depicts the correlation of PLA1A with common diagnostic markers of MM. The relative PLA1A levels are positively correlated to S-100 and HMB-45 (*r* = 0.293 and *r* = 0.353 respectively, both *p* ≤ 0.001; Fig. 2B,C). By simultaneously considering both tissue and serum levels of PLA1A, the results revealed that PLA1A was inversely correlated to Melan-A (*r* = − 0.025, Supplementary Fig. 1A). Strikingly, PLA1A was not correlated to the expression of Ki67 (*r* = 0.033, *p* = 0.133; Supplementary Fig. 1B) and TP-53 (*r* = 0.01, *p* = 0.245; Supplementary Fig. 1V). In general, higher PLA1A levels were correlated with disease severity and metastatic lesions among patients with melanoma.

Previous studies demonstrated that patients with BRAF/NRAS-mutant exhibit enhanced invasive characteristics, which are correlated to disease severity^10,14^. Therefore, in order to evaluate the value of PLA1A expression as a diagnostic marker in the treatment of BRAF/NRAS-mutant melanoma, the expression of PLA1A in BRAF and NRAS mutant samples was evaluated through the use of the linear logistic regression in Fig. 2D,E, respectively. The results showed that PLA1A was positively correlated to the frequency of BRAF and NRAS (*r* = 0.136,* p* = 0.01;* r* = 0.065,* p* = 0.059, respectively). In total, the positive correlation of PlA1A with BRAF and NRAS led to the hypothesis that PLA1A might be a potential diagnostic marker in BRAF/NRAS-driven melanogenesis.

### PLA1A expression as a prognostic marker in BRAF/NRAS-mutated samples of melanoma

**Figure 3:**
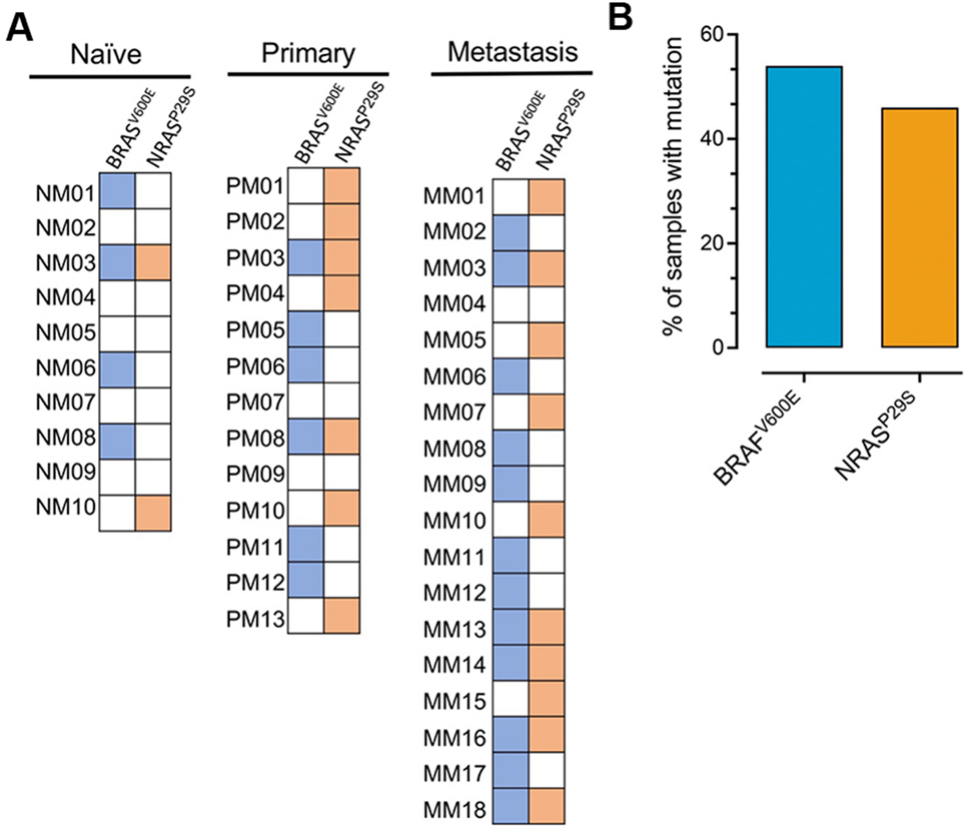
BRAF- and NRAS-mutant melanoma rates among different study groups. (**A**) Summary of the genetic landscape of BRAF V600E and NRAS P29S mutant genes in normal naïve/normal melanoma (n = 10), primary melanoma (n = 13) and metastasis melanoma (n = 18). (**B**) Bars represent the percentage of somatic BRAF V600E and NRAS P29S mutations, distinguished by their individual colors.

It is clear that a specific combination between BRAF and NRAS mutations leading to up- or downregulation of novel diagnostic marker in MM may be more consistent with the diagnosis of advanced stage III melanoma^30,31^. Therefore, the diagnostic value of PLA1A in BRAF and NRAS-mutated samples of melanoma patients was investigated to confirm the diagnostic utility of PLA1A during melanomagenesis. The *BRAF*^*V600E*^ and *NRAS*^*P29S*^ mutation status of each tumor was determined using Sanger sequencing as previously described (Fig. 3A)^32–34^. Overall, 54% of the tumors in the discovery cohort had canonical *BRAF*^V600E^ mutation, and 46% were *NRAS*^*P29S*^ (Fig. 3B). Interestingly, the proportion of *BRAF*^V600E^ (12 of 22) and NRAS-mutants (10 of 19) was higher in the metastatic group compared to the one in the naïve/control group (*p* ≤ 0.001) (Table 2). These findings confirmed that high BRAF/NRAS-mutated tumors were associated with a more severe stage of melanoma. A significant relationship was found between advanced melanoma stages and increased BRAF/NRAS mutation rate (*p* ≤ 0.001).

**Table 2:** BRAF and NRAS mutation variables in study patients.

Variable	Control group	Melanoma patients		Disease-free survival^a^		
	Naïve (n = 10)	Primary (n = 13)	Metastasis (n = 18)	Pooled sensitivity	Pooled specificity	AUC
*BRAF*^*V600E*^ mutation n (%)				0.623	0.617	0.75
Negative	6 (60)	7 (53.8)	6 (33.4)			
Positive	4 (40)	6 (46.2)	12 (66.6)**^,#^			
*NRAS*^*P29S*^ mutation n (%)				0.555	0.553	0.61
Negative	8 (80)	6 (46.2)	8 (44.4)			
Positive	2 (20)	7 (53.8)**	10 (55.5)**^,#^			

Data are presented as mean ± SD for all others. Naïve, healthy subjects with normal skin.

*BRAF V600E* B-Raf proto-oncogene, serine/threonine kinase with valine‐to‐glutamic acid substitution at position 600, *NRAS P29S* Neuroblastoma RAS viral [v-ras] oncogene homolog with proline-to-serin acid substitution at position 29.

*p < 0.05 and **p < 0.001 vs the naïve group; ^#^*p* < 0.05 and ^##^*p* < 0.001 vs the primary group.

^a^The disease-free survival (DFS) was analyzed using the Kaplan–Meier method, log-rank test, and Cox proportional hazard model. The area under the curve (AUC) of receiver operating characteristic (ROC) an analyzed by using spearman’s rank correlation coefficient test helped determine the relationship between two sensitivity (ordinate) and 1-specificity (abscissa).

**Figure 4:**
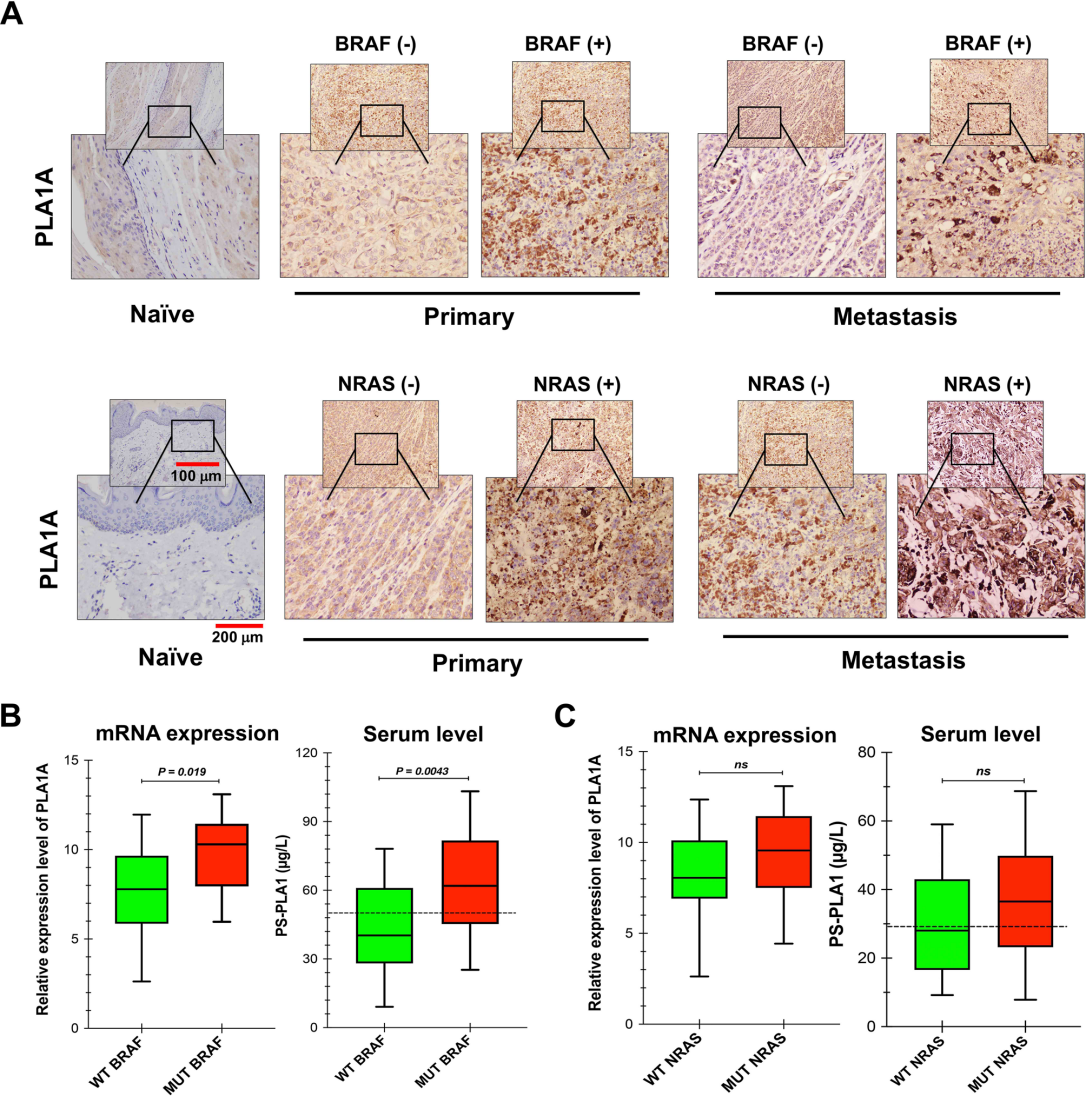
PLA1A expression in BRAF- and NRAS-mutant subjects with melanoma. (**A**) Gallery of representative immunostaining of PLA1A expression in BRAF-WT/MUT type (orange) and NRAS-WT/MUT among naïve, primary, and metastatic groups. Patents with wild and mutant type are represented by the blue and orange box, respectively. (**B**) The histogram shows the frequency of PLA1A mRNA expression and serum levels between BRAF-WT (green) and BRAF-MUT (Red) type of patients. (**C**) Distribution of PLA1A expression in patients with NRAS-WT (green) and NRAS-MUT (Red) type.

PLA1A expression in BRAF-WT/MUT type and NRAS-WT/MUT type by immunohistochemistry is shown in Fig. 4A. Morphometrically, all high-grade nodular MM samples, which were categorized as BRAF/NRAS-MUT, showed high PLA1A expression with significant intensity. Additionally, PLA1A expression was enclosed in the well-circumscribed foci of BRAF^+^ samples in both metastatic and primary samples, which were analyzed over the whole sections. In contrast, PLA1A was highly distributed over the entire tumor section and not just in the focal positive section. In addition, a significant difference in the increased PLA1A positive cells was found between advanced BRAF/NRAS-MUT melanoma stages and naïve/control melanoma samples. By considering both PLA1A mRNA and serum levels simultaneously, PLA1A levels were significantly increased in BRAF/NRAS^+^ melanoma patients (*p* ≤ 0.05; Fig. 4B,C). The ratio of PLA1A was significantly higher in patients possessing the BRAF-MUT (*p* = 0.019 for PLA1A mRNA and *p* = 0.004 for serum PLA1A levels; Fig. 4B) compared to the BRAF-WT patients. Similar to the results from BRAF^+^ melanoma patients, serum PLA1A levels were increased in MUT-NRAS melanoma patients compared to the level in the WT-NRAS (36.25 ± 16.24 µg/L vs 29.56 ± 14.26 µg/L), although this difference was not statistically significant (Fig. 4C; *p* > 0.05). These results suggested that enhanced PLA1A mRNA and serum levels are significantly present in BRAF^+^ melanoma patients, compared to NRAS^+^ melanoma patients. Interestingly, our results indicated that both BRAF^+^ and NRAS^+^ MM patients had high PLA1A expression compared to the expression in primary and naïve/control patients (Supplementary Fig. 2). In general, these findings suggested that PLA1A could be considered as a potential diagnostic marker in BRAF-driven melanomagenesis.

### Diagnostic value of PLA1A in advanced MM

**Figure 5:**
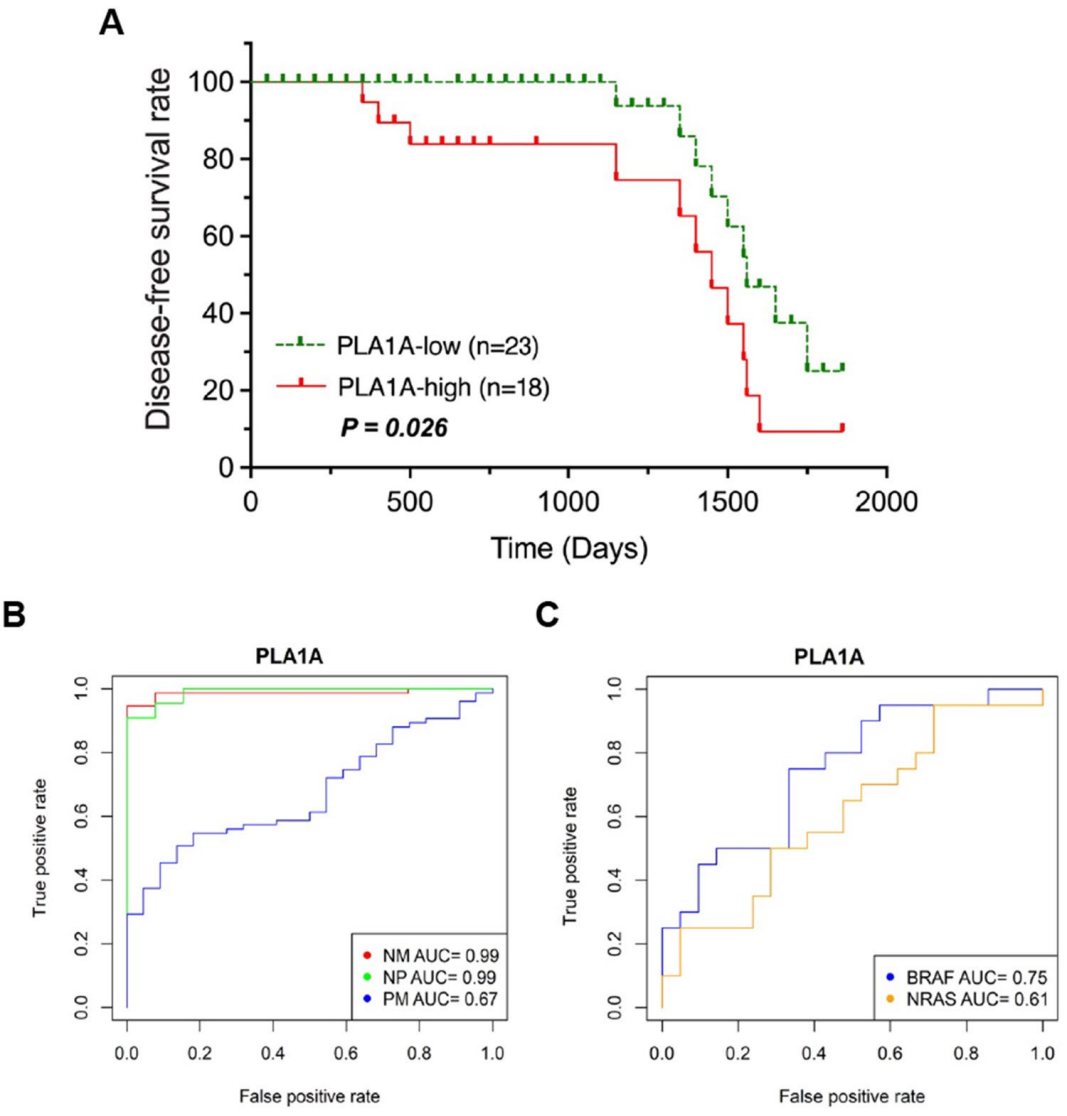
Diagnostic value of PLA1A in advanced metastasis melanoma. (**A**) The disease-free survival (DFS) rate for PLA1A expression in 23 patients with low-PLA1A and 18 patients with high-PLA1A. The patients were categorized as either low-PLA1A when FC > 0.072 for PLA1A mRNA expression and > 40.91 µg/L for serum PLA1A levels. The high-PLA1A is FC ≥ 0.072 for PLA1A mRNA expression and ≥ 40.91 µg/L for serum PLA1A levels. (**B**) Receiver operating characteristic (ROC) curve for PLA1A expression between normal naïve/metastasis melanoma group (red line), naïve/primary melanoma (green line), and primary/metastasis melanoma (blue line). (**C**) ROC curve to determine the optimal cut‐off value of PLA1A to distinguish melanoma patients with BRAF-mutation (blue line) and NRAS-mutation (orange line). The diagnostic values were calculated by considering both PLA1A mRNA expression and serum levels.

Finally, our patients were categorized into groups according to their PLA1A levels as lower than, equal to, or higher than the median expression of PLA1A (FC: 0.072 for PLA1A mRNA expression and 40.91 µg/L for serum PLA1A levels) to determine any possible relationship between PLA1A expression and disease-free survival (DFS) of melanoma patients using the Kaplan–Meier method and log-rank test (Fig. 5A). By considering both PLA1A mRNA and serum levels, 56% (23 of 41 patients) of the patients were categorized as either low-PLA1A (FC > 0.072 for PLA1A mRNA expression and > 40.91 µg/L for serum PLA1A levels), while almost 44% (18 of 41 patients) of the patients were categorized as a high-PLA1A (FC ≥ 0.072 for PLA1A mRNA expression and ≥ 40.91 µg/L for serum PLA1A levels). As expected, melanoma patients with PLA1A1-high tumors had significantly shorter DFS compared to that in patients with PLA1A-low tumors (5-year DFS rate: 69.3% vs 75.4%, *p* = 0.026) (Fig. 5A). This result indicated that the prognosis of patients with high-PLA1A tumors was worse compared to that of patients with low-PLA1A tumors (*p* < 0.05, Fig. 5A). The correlation between different stages of melanoma and 5-year DFS is shown in Fig. 5B. The area under the curve (AUC) of the receiver operating characteristic (ROC) analysis of naïve/primary, naïve/metastasis, and primary/metastasis group was 0.99, 0.99, and 0.67, respectively. The analysis based on specimen types indicated that PLA1A had a relatively accurate diagnostic value in discriminating the naïve melanoma samples from advanced melanoma samples, with a sensitivity of 0.91 and specificity of 0.57 (Fig. 5B and Supplementary Fig. 3). By considering both PLA1A mRNA and serum levels, the univariate analysis among BRAF/NRAS mutated samples demonstrated that PLA1A was more accurate for BRAF-MUT samples compared to NRAS-MUT samples (Fig. 5C). As shown in Fig. 5C and Supplementary Fig. 3B, the pooled sensitivity and specificity were higher in the BRAF-MUT samples compared to NRAS-MUT samples (Table 2). Moreover, PLA1A had the highest sensitivity, specificity, and AUC in BRAF-MUT advanced melanoma samples, suggesting that PLA1A could be used as a marker for an effective prediction and diagnosis of advanced BRAF-mutant melanoma cancer.

## Discussion

Our results revealed for the first time that the upregulation of PLA1A in the serum of advanced metastatic melanoma tissues represent an excellent diagnostic marker in BRAF/NRAS-driven melanomagenesis. Besides, the upregulation of PLA1A mRNA in tissue samples of advanced MM patients represents a more accurate diagnostic marker. A significant positive correlation was found between PLA1A and disease severity, as well as routine diagnostic markers such as HMB-45, S-100 and Ki-67, which supports the idea that PLA1A expression could be correlated to diagnostic markers in MM, and is involved in melanogenesis among advanced melanoma human subjects. Therefore, during the melanogenesis, the serum PLA1A level could be used as a non-invasive marker in the diagnosis of MM. The evidence to consider PLA1A as a diagnostic marker were shown, which could help to distinguish naïve melanoma samples from advanced melanoma samples. These findings suggested that PLA1A could be considered as a clinical marker for an accurate prediction and diagnosis of advanced BRAF-mutant melanoma cancer.

Reliable diagnostic markers and the assessment of the molecular status of the tumor in the patients are required for an effective targeted therapy in the treatment of high TMB melanoma, leading to an approved adjuvant therapy that can be used among patients with advanced MM^11,12,20^. To the best of our best knowledge, we are the first showing that current serological and immunohistochemical markers in melanoma had significantly limited the detection of the advanced stage of this disease, which metastasized from the primary site^35–37^. Among solid advanced melanoma tumors, it is difficult to find the markers precisely classifying the stage of melanoma, resulting in the currently known sensitivity and resistance to therapeutic agents of specific mutated patients. Furthermore, most of the serological and histological diagnostic markers of melanoma are based on the detection of melanocytes, rather than melanoma itself^37^. Likewise, BRAF/NRAS mutant melanoma tumors contain several high-frequency driver mutations, and therefore represent a big challenge to dermatologists or oncologists in the discovery of unique and stable upregulated oncogenic markers among highly invasive melanoma samples for an early diagnosis and targeted therapy^19,38,39^. Therefore, novel markers are mostly based on genomic markers of carcinogenetic gene mutations^40^. Accordingly, MM patients that are positive for BRAF/NRAS mutation increased their survival with treatments involving novel therapeutic markers^39,40^.

The serum PLA1A level is associated not only with tumorigenesis, but also to most inflammatory diseases, including systemic lupus erythematosus, hepatitis, and hyperthyroidism^41–43^. Extensive evidence demonstrated that PLA1A regulates different stages of carcinogenesis associated with angiogenesis, differentiation, proliferation, invasion, apoptosis, and metastasis^44^. PS-PLA1, the sole substrate of LysoPS, is detectable among several physiological conditions and is normally restricted to the inner surface of the cell membrane, apoptotic cells, antigen-activated lymphocytes and immunological escape of melanoma cells^45^. It is clear that the cause of MM is mostly related to mutagenesis, with the involvement of several oncogenes, including *BRAF* and *NRAS*^40^. Thus, in this work, PLA1A was evaluated for the first time among the histopathological subtypes of the BRAF/NRAS-driven melanoma cancer population. As no therapeutic agents have been approved specifically for NRAS-mutant melanoma, our aim was to compare the benefit of PLA1A in the BRAF- and NRAS-driven melanoma cancer population^39,46^. Our results showed that high serum PLA1A levels were associated with tumors invasion, as well as a higher incidence of *BRAF*^*V600E*^ and *NRAS*^*P29S*^ mutation metastasis. Interestingly, V600E hotspot mutations were significantly overexpressed among subcutaneous metastases. Thus, it is possible that V600E hotspot mutation on *BRAF* leads to more than one *NRAS*^*P29S*^ mutation, which can induce different physiological functions in response to LysoPS, leading to increased serum PS-PLA1 levels in melanoma patients. Furthermore, the expression of LysoPS receptors differs among BRAF-mutant melanoma samples, as well as in other broad perspective of TMB-encompassing cells, immunomodulatory cells, and other cell types^47^. Prospectively, further functional studies on the effect of PS-PLA1A/LysoPS receptor axis on melanogenesis could additionally confirm PLA1A as a potential therapeutic target for different types of TMB-driven melanomagenesis.

In addition, S-100 and HMB-45 are excellent immunohistochemical markers with a sensitivity and specificity of 100% in distinguishing melanoma from non-melanocytic carcinomas^48,49^. In this regard, our present work demonstrated that the relative PLA1A levels were positively correlated to both S-100 and HMB-45 markers. Because of the poor 5-year survival rate and prognosis of patients with late stage melanoma, PLA1A, in conjunction with S-100 and HMB-45, could serve as a novel marker to distinguish lymph node naive melanoma from melanoma metastasis^49^. However, a further clinical confirmation is needed to determine the diagnostic and prognostic value of PLA1A levels in melanoma, as well as to establish which patients need more aggressive therapy^50^.

As regard the prototypic testing of advanced cutaneous melanoma, PLA1A increased the hope in finding effective clinical markers for high TMB, and a potential cure^15,29^. Interestingly, this is the first report demonstrating that PLA1A mRNA expression, with a cut-off value of 0.072 and serum PLA1A levels of 40.91 µg/L could be used to discriminate naïve melanoma samples from advanced melanoma samples, with a sensitivity of 91% and a specificity of 57%. Previous studies proposed that the relatively low specificity of PS-PLA1 may be due to the increased serum PS-PLA1 levels in control patients, although these are still lower compared to those observed in patients with primary melanoma^28,42^. Importantly, the high sensitivity, specificity, and AUC in BRAF-MUT advanced melanoma samples suggested that PLA1A could be used as a marker for an effective prediction of advanced BRAF-mutant melanoma cancer. Therefore, the overall diagnostic utility of the measurement of PLA1A mRNA expression or serum levels in BRAF-mutant MM was unsatisfactory. Whether or not it is combined with other markers, the diagnostic value of PLA1A as an innovative therapeutic target combined with BRAF inhibition requires further elucidation.

Our finding showed for the first time that PLA1A with the highest sensitivity, specificity, and AUC could be used as a single indispensable marker for diagnosis, screening, and prognosis in BRAF-MUT advanced melanoma. Similarly, our results proposed to combine PLA1A with other routine invasiveness diagnostic markers of melanoma: HMB-45, S-100 and Ki-67, with the hope that these markers, after proper standardization, might serve as a potent indicators in the prognosis and determination of the metastatic stage of melanoma^51^. In precision oncology, real-time molecular monitoring of diagnostic markers is used to identify patients with a different stage, but it should also play a critical role in finding markers that predict the treatment outcome in patients with different molecular classification and specific subtype^52^. In this regards, single-site biopsy of PLA1A could be considered as a diagnostic marker of BRAF-mutant metastasis in melanoma cancer, since it is sufficiently sensitive and precise in distinguishing melanoma patients with BRAF-mutated and NRAS-mutated advanced melanoma. Thus, investigating multiple mRNA marker profiles would be beneficial to improve the sensitivity of PLA1A as a single diagnostic marker or as a novel component in the immunohistochemical panel of metastatic melanomas. Surely, this experimental research should be followed by a further work to characterize the clinical applicability of PLA1A, which is actually underway.

These results from this study clearly adhere to the idea that PLA1A is a potential diagnostic and significant therapeutic marker among advanced MM. These results point to the probability that PLA1A imbalance and clarification of its characteristics can help to recognize the pathogenesis of melanoma, which may be partially due to the serum and lung tissue microenvironment. A small sample size, less functional studies, and less homogeneous distribution of samples based on phytology and mutation parameters might be a springboard for large sample size with well-pathologically characterized melanoma samples in future studies. The clinical use of PLA1A, a novel therapeutic target of MM progression, requires further studies that are currently ongoing in our laboratory.

In conclusion, our results showed that PLA1A might be a promising diagnostic marker in patients with advanced metastatic melanoma, providing a more accurate diagnostic marker for BRAF-mutant samples of MM patients. A significant positive correlation was found between PLA1A and disease severity as well as diagnostic markers, thus PLA1A could be used in the future as a non-invasive marker in the diagnosis and therapy of metastatic melanomas. Further well-designed cohort studies are needed to establish the clinical significance of PLA1A for personalized MM diagnosis.

## Methods

### Ethic statement

This study was approved by the “Ethics Review Board” at the Affiliated Hospital of Southwest Medical University (No. KY2019041). Written informed consent in agreement to the requirement of the Declaration of Helsinki (1983 Revision) was obtained from all participants or their guardians prior to the study to use their clinical and pathology information, as well as for mutation analysis. The volunteers were informed about the goal and protocols of the study. Additionally, all clinical assessments were performed according to the local Ethics Committee guidelines of the Pathology Department and Oncology Department at The Affiliated Hospital of Southwest Medical University in Luzhou, Sichuan, China.

### Patient population and clinical assessment

Patients with melanoma that were preliminarily selected for this prospective study were confirmed as having the disease by two expert oncologists (S.I. and Q.L.W.), as well as one pathologist (C.Z.). All participants were adult melanoma patients who were referred to the Department of Dermatology and Oncology of the Affiliated Hospital of Southwest Medical University, Luzhou, China from April 2020 to September 2020. Melanoma was diagnosed according to the World Health Organization (WHO) guidelines and the pTNM Union for International Cancer Control (UICC) pathological staging criteria. All patients were treated at the Department of Dermatology and Oncology, the Affiliated Hospital of Southwest Medical University, Luzhou, China. The samples were collected from naïve/normal melanoma subjects with stage 0–I and primary melanoma subjects with stage II–III before the treatment, or subjects with stage III–IV melanoma when they did not receive any treatment except palliative care. The demographic and histopathologic variables of all subjects, including medical, reproductive and family history, tumor site, histological type, treatment and survival were correctly recorded in a database. Metastasis was assessed by fluorine-18-fluorodeoxyglucose positron emission tomography/computed tomography (^18^F-FDG PET/CT) imaging. All metastatic organs were evaluated and confirmed by two expert pathologists (C.Z. and Q.L.W.). In addition, laboratory tests including blood routine, and liver function tests were performed in patients who need a confirmation for the presence of metastases. All patients with MM were clinically stable and had not experienced any malignancy for ≥ 2 months prior to the inclusion in the study. Participants with some autoimmune disorders and systemic inflammatory disorders such as type 1 diabetes mellitus, interstitial lung disease, rheumatoid arthritis, or other immune related diseases were excluded. In addition, history of acute bronchiolitis/pneumonia, systemic lupus erythematosus, thyroid disorders, multiple metastatic sites, and/or participation in simultaneous clinical trials were considered as exclusion criteria. Disease stages were classified according to the AJCC 8th edition^53^. All patients underwent standard treatments based on the practice of each treating physician. The demographic and baseline clinico-philological characteristics of patients are listed in Table 1.

### Serum samples

The serum samples used in this study were residuals samples that were obtained from all subjects when they did not undergo any treatment, with the exception of palliative care. To obtain the serum samples, 2 mL blood were left to clot at room temperature for at least 30 min, and then centrifuged at 1200×*g* for 8 min. The serum was collected and subsequently divided into 500 µL aliquots, which were then stored at − 80 °C until further DNA and RNA extraction.

### Mutation analysis

In order to detect V600E mutation in *BRAF* gene (c.1796T > A) and P29S in *NRAS* gene (c.85C > T), DNA was isolated from 5 μm-thick sections of all tissue samples through the use of QIAamp DNA FFPE Tissue kids, according to the manufacturer’s protocol. These recurrent mutations in *BRAF* and *NRAS* genes were the most common genetic alterations in melanoma, and were identified via qRT-PCR through the use of specific hybridization probes that allow the detection of the following variants^9,13,14^. Primer sequences and specific PCR reactions are available on Supplementary Table 1. The concentration of extracted DNA was measured using a NanoDrop2000 spectrophotometer (Thermo Scientific, Wilmington, DE, USA). The optical density ratio 260/280 ~ 1.8 and 260/230 > 1.5 was assessed to evaluate the quality. The detection of variants was performed using LightCycler 96 real-time PCR machine, according to standard protocols as previously described^54,55^. PCR products were confirmed via Sanger sequencing methods on an ABI-3500 sequencer (Applied Biosystems Inc., Foster City, CA, USA). Finally, the resulting sequences were compared to consensus sequences via Seqman software (Lasergene 8.0; DNASTAR, Inc., Madison, WI). All reactions were performed with two replicates per sample, as well as a non-reverse transcription control and non-template control for each test.

### Quantitative real-time PCR analysis

In order to quantify and compare the expression of *PLA1A1* gene with the *NRAS* and *BRAF* genes, qRT-PCR was performed based on the standard protocols as previously described^56^. In brief, total RNA was extracted from frozen biopsies using Trizol reagent (Takara, Dalian, China), according to the manufacturer's instructions. The expression of target genes was quantified using predeveloped TaqMan assays from Applied Biosystems (Life Technologies, Foster City, CA) and the expression was normalized to that of the *18SRNA* housekeeping gene using the comparative Ct method^57,58^. Primer sequences used in this investigation are listed in Supplementary Table 1.

### ELISA measurements

Serum levels of human PLA1A were measured using the standard specific double-antibody sandwich ELISA kits, according to the manufacturer’s instructions among three study groups (1:1000 dilution; ABIN5072368, Biotech Co. Technology, Beijing, China)^26,27^. The lower limit of detection of each kit (supplier's data) was 18.75 pg/mL. The ELISA reader and washer were Stat-Fax 2100 and Stat-Fax 2600 (Awareness Technologies, FL, USA), respectively.

### Immunohistochemistry

The best frozen sample was used for hematoxylin and eosin analysis, as well as histological conformation. Immunostaining of PLA1A, S-100, HMB-45, Melan-A, Ki-67, and P53 was performed using the streptavidin–biotin alkaline phosphatase complex method by the Vectastain ABC-AP standard kit (Vector Laboratories; Burlingame, CA) as previously described^59,60^. In brief, antigen retrieval was achieved by heating the 5 μm-thick paraffin-embedded sections in a high-temperature pressure cooker for 90 s in citrate buffer at pH 6.0. Then, endogenous peroxidase activity was suppressed by the treatment with methanol + 1% hydrogen peroxide for 30 min at room temperature. Nonspecific reactions and quench endogenous alkaline phosphatase were blocked using 2% normal mouse serum for 30 min at RT (Santa Cruz Biotechnology, Santa Cruz, CA) and levamisole (Vector Laboratories, Burlingame, CA), respectively. The slides were incubated using the primary monoclonal antibodies of the proteins mentioned above, based on the related concentration (see Supplementary Table 2). The endogenous peroxidase activity was quenched in 0.5% H_2_O_2_ for 10 min, and the slides were incubated with primary monoclonal antibody, according to the manufacturer's guidelines. Subsequently, the slides were incubated in a humid chamber for 10 min at RT with biotinylated secondary antibody, according to the manufacturer’s instructions (Vector Laboratories; Burlingame, CA). Then, an avidin–biotin alkaline phosphatase complex was added for 30 min at RT (3 µl 1:400 Promega, Madison, WI), and the color was developed using the Vector Red alkaline phosphatase substrate kit (Vector Laboratories). Additionally, normal mouse IgG, normal preimmune rabbit IgG, or Tris-buffered saline was used as negative control. The patients were coded, and measurements were made in a blinded mode by two expert pathologists (S.Y. and C.Z.). The Ki-67, P53 and S-100 labeling index were calculated on five randomly selected fields of each tumor sample as number of the invasive index positive cells/total counted cells at 400× magnification. In addition, five high power visual fields (400×) were randomly selected for each section to calculate the percentage of area with a positive expression of HMB-45 and Melan-A. The proportion of HMB-45 and Melan-A positive expression was calculated as area of HMB-45 and Melan-A positive staining/the total area under a high magnification field of vision. Since PLA1A staining is mainly observed in the cytoplasm of cancer cells, the immunostaining intensity of PLA1A was classified into three grades: negative-to-weak, moderate, and strong. Sections where more than 30% of cells were scored as negative-to-weak were classified as PLA1A-negative, and those with 30% or less were moderately classified as PLA1A-low, and 40% of all samples that had strong expression of PLA1A immunostaining were classified as PLA1A-high. All these counts were performed in a blinded manner. All sections were visualized using a Zeiss Axioplan 2 microscope, and images were captured on a Windows NT workstation and analyzed using Zeiss Axiovision software (Zeiss; New York, NY).

### Statistical analysis

Statistical analysis was performed using SPSS software version 21.0 (Chicago, Illinois, USA). All tests were repeated three times or more. Results were presented as mean ± Standard Deviation (SD) or median (range). Serum PLA1A levels were compared among groups using the one-way Kruskal–Wallis test. If the results of this test indicated significance, a Mann–Whitney test was used for post-hoc analysis to compare two groups, with corrections of the P-values conducted according to Bonferroni. A Spearman’s rank correlation coefficient test was employed to determine the association between two variables. Linear stepwise multivariate regression was performed to identify parameters that correlated with serum PLA1A levels. Furthermore, DFS was analyzed using the Kaplan–Meier method, log-rank test, and Cox proportional hazard model. In order to identify the cut-off threshold effects and construct the ROC curves, spearman’s rank correlation coefficient test was used to determine the relationship between two sensitivity (ordinate) and one specificity (abscissa)^61^. The results were considered statistically heterogeneous when *p* < 0.05 and/or I^2^ > 50%^62^. The diagnostic threshold effect was analyzed using the Spearman correlation coefficient test. In all tests, two-sided *p* values less than 0.05 were considered statistically significant. All graphs were designed using GraphPad Prism software 5.0 for windows (GraphPad Software Inc., La Jolla, CA, USA).

## Supplementary Information

Supplementary Information.

## Data Availability

All data associated with this study are present in the paper or the Supplementary Information. Microarray datasets were submitted to Gene Expression Omnibus (GEO) public repository and data can be accessed through accession number GSE34460, GSE18509, GSE24996, GSE7553, GSE15605, and GSE19234. Source data are provided in this article^21^.
