# Supplementary material for: PLA1A expression as a diagnostic marker of BRAF-mutant metastasis in melanoma cancer
Source: Sci Rep. 2021 Mar 15;11:6056. doi: 10.1038/s41598-021-85595-7 (PMC7961027; doi:10.1038/s41598-021-85595-7)
Supplement: Supplementary file 1 — Supplementary Information. [file 41598_2021_85595_MOESM1_ESM.pdf]

Supplementary Information

PLA1A expression as a diagnostic marker of BRAF-mutant metastasis in melanoma cancer

Gang Yang[1,2,†], Shuya Liu[1,†], Mazaher Maghsoudloo[3,4,†], Marzieh Dehghan Shasaltaneh[5], Parham Jabbarzadeh Kaboli[6,7], Cuiwei Zhang[8], Youcai Deng[9], Hajar Heidari[4], Maliheh Entezari[4], ShaoZhi Fu[1], QingLian Wen[1,*] & Saber Imani[1,*]

[1]Department of Oncology, The Affiliated Hospital of Southwest Medical University, Luzhou, Sichuan, China
[2]Department of Oncology, Anyue Hospital of Traditional Chinese Medicine, Second Ziyang Hospital of Traditional Chinese Medicine, Ziyang, Sichuan, China
[3]Laboratory of Systems Biology and Bioinformatics, Institute of Biochemistry and Biophysics, University of Tehran, Tehran, Iran
[4]Department of Genetics, Faculty of Advanced Science and Technology, Tehran Medical Sciences, Islamic Azad University, Tehran, Iran
[5]Department of Biology, Faculty of Science, University of Zanjan, Zanjan, Iran
[6]Department of Pharmacology, School of Pharmacy, Southwest Medical University, Luzhou, Sichuan, China
[7]Graduate Institute of Biomedical Sciences, Research Center for Cancer Biology, and Center for Molecular Medicine, China Medical University, Taichung, Taiwan
[8]Department of Pathology, The Affiliated Hospital of Southwest Medical University, Luzhou, Sichuan, China
[9]Institute of Materia Medical, College of Pharmacy, Army Medical University (Third Military Medical University), Chongqing, China
* Corresponding. wql73115@hotmail.com (Q.L.W.); saberimani@swmu.edu.cn (S.I.)
† These authors contributed equally to this work

Inventory of supplementary information

- Supplementary Table 1 (Page 1)
- Supplementary Table 2 (Page 2)
- Supplementary Fig. 1 (Page 3)
- Supplementary Fig. 2 (Page 4)
- Supplementary Fig. 3 (Page 5)

Supplementary Table 1. List of specific primers sequences for qRT-PCR and PCR of candidate variants.

Target gene	Accession No.*	Sequence (5'→3')	Length	T_m	GC%
PLA1A	NM_001206960.2	F, CAGACACCGACAATTTGGGTA	21	57.64	45.00
		R, CCAGGGCGCTGATGTAGAG	19	58.58	50.00
RAC1 (P29S)	NM_000546.6	F, TGCTAACACCGGGTACCTAAAC	22	59.02	50.00
		R, TCATCCAGTCTCTGTACCTCAC	22	59.02	55.00
BRAF (V600K)	NM_001354609.2	F, AGTAGGCTTGGCATGACAGT	20	59.01	50.00
		R, TGGTTGGAATCTGCTCGTCT	20	59.02	50.00
B-active	NM_001101	F, GGAGATTACTGCCCTGGCTCCTA	23	55.09	45.00
		R, GACTCATCGTACTCCTGCTTGCTG	24	57.46	50.00

Abbreviations: PLA1A, phosphatidylserine-specific phospholipase A1- alpha (PLA1A); BRAF, B-Raf proto-oncogene, serine/threonine kinase; RAS, Neuroblastoma RAS viral [v-ras]; F, forward primer; R, reverse primer; Tm, optimum primer melting temperatures; GC%, guanine and cytosine percentage.
*Genbank accession number of cDNA and corresponding gene, available at http://www.ncbi.nlm.nih.gov/

Supplementary Table 2. List of specific antibodies for immunohistochemistry and western blot.

Epitope	Spices	Company	Catalog No.	Dilation	Source
		Primary antibodies			
S-100	Human, Mouse	eBioscience	14-5785-81	1:2000	Mouse
HMB-45	Human, Rabbit	Santa Cruz	SC-25388	1:1000	Rabbit
Melan-A	Human, Mouse	Santa Cruz	SC-271984	1:2000	Mouse
Ki-67	Human, Mouse	Abcam	ab175430	1:5000	Mouse
P53	Human, Mouse	Santa Cruz	SC-166476	1:3000	Mouse
PLA1A	Human, Mouse	Abcam	ab233145	1:1000	rabbit
		Secondary antibodies or conjugates			
anti-mouse HRP	Mouse	Santa Cruz	G3114	1:5000	Goat
anti-rabbit HRP	Rabbit	Bioworld Technology	A0208	1:3000	Goat

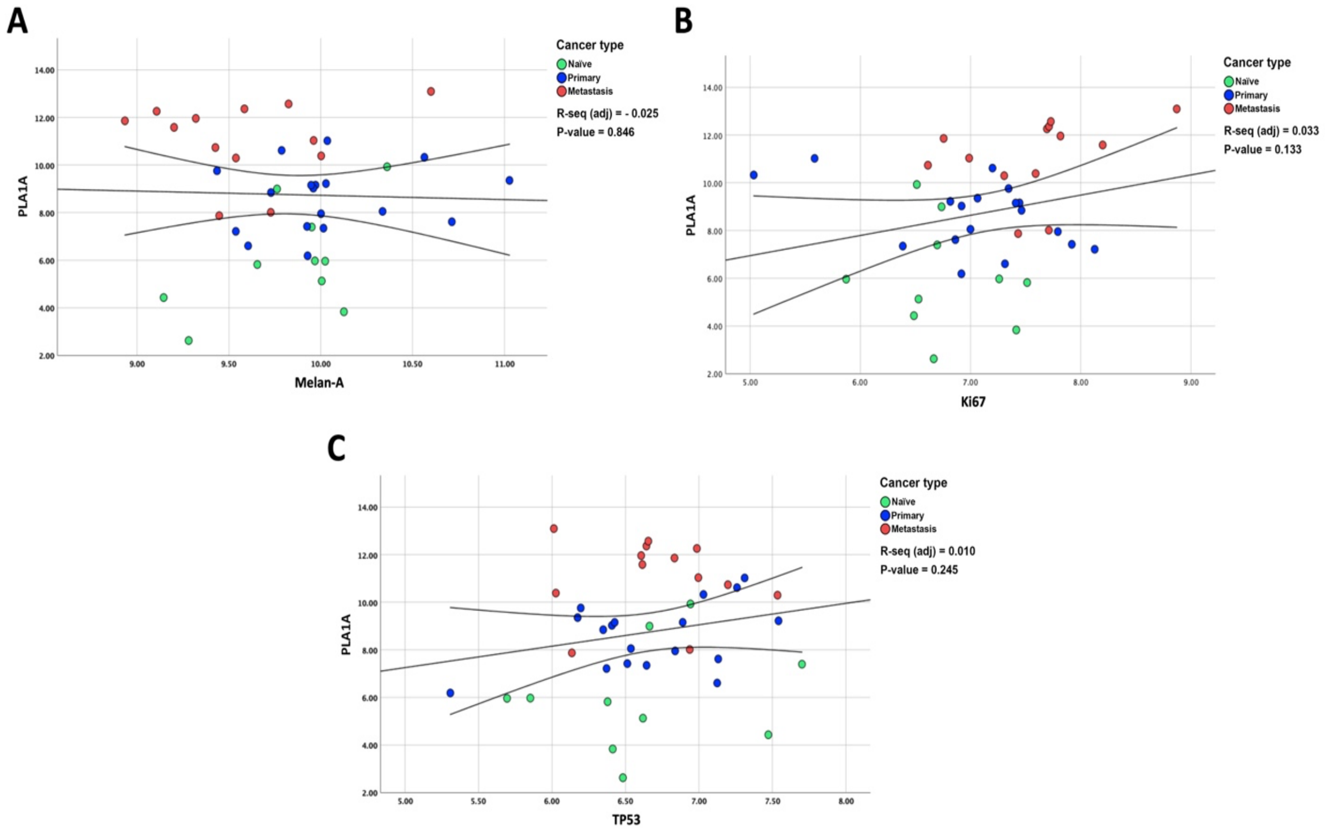

Supplementary Fig. 1 Correlation coefficient of PLA1A with metastatic melanoma biomarker. The linear logistic regression analysis was performed to predict the accuracy of the detection of PLA1A expression by considering both tissue and serum levels of PLA1A.

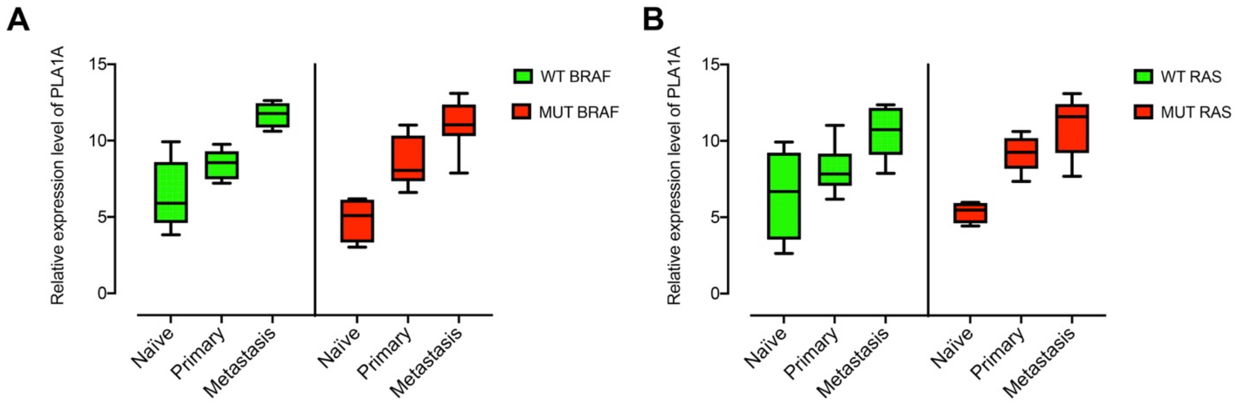

Supplementary Fig. 2 a-b Comparing the PLA1A expression in patients possessing the BRAF-MUT and NRAS- MUT between naïve/normal melanoma, primary melanoma, and metastasis melanoma groups.

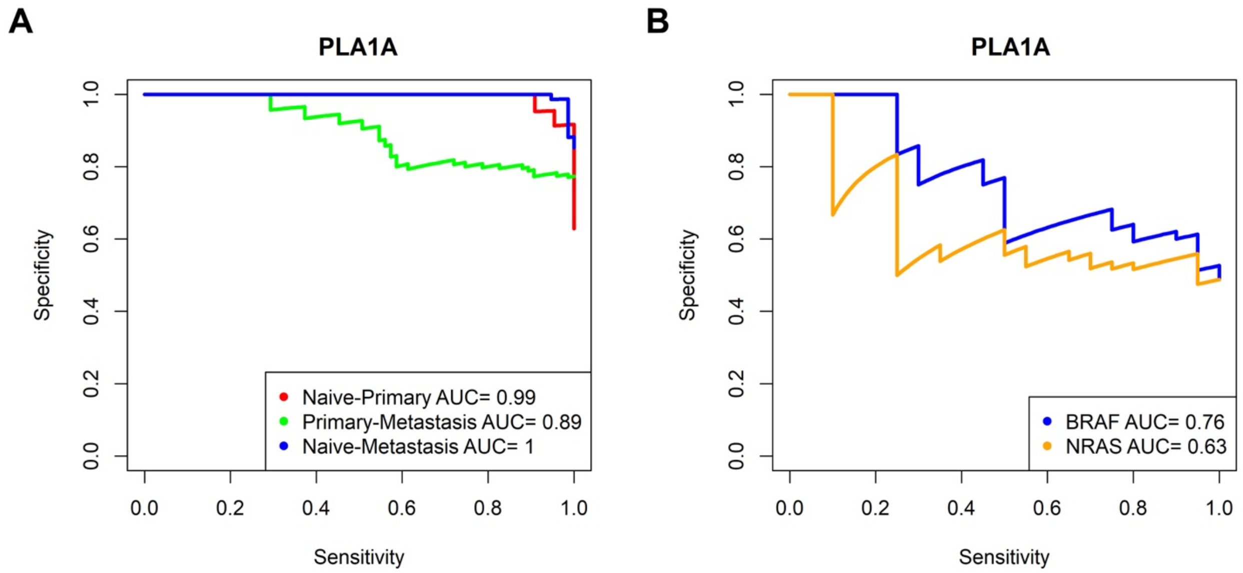

Supplementary Fig. 3 a-b The pooled sensitivity and specificity of PLA1A in BRAF/NRAS mutated samples. The univariate analysis among BRAF/NRAS mutated samples were performed by considering both tissue and serum levels of PLA1A.
